# Chronological dataset of soil respiration fluxes from a seasonally dry forest in Northwest México

**DOI:** 10.1016/j.dib.2023.109716

**Published:** 2023-10-23

**Authors:** Martha L. Vargas-Terminel, Dulce Flores-Rentería, Zulia M. Sánchez-Mejía, Nidia E. Rojas-Robles, Maritza Sandoval-Aguilar, Bruno Chávez-Vergara, Agustín Robles-Morua, Jaime Garatuza-Payan, Enrico A. Yépez

**Affiliations:** a Departamento de Ciencias del Agua y Medio Ambiente, Instituto Tecnológico de Sonora, Ciudad Obregón, Sonora 85000, México; b Departamento de Sustentabilidad de los Recursos Naturales y Energía, CONACYT-Centro de Investigación y de Estudios Avanzados del IPN Unidad Saltillo, Ramos Arizpe, Coahuila 25900, México; c Department of Environmental Sciences, University of California Riverside, Riverside, CA 92521, USA; d Instituto de Geología, Universidad Nacional Autónoma de México, Ciudad de México 04510, México; e Laboratorio Nacional de Geoquímica y Mineralogía, Universidad Nacional Autónoma de México, Ciudad de México 04510, México; f Laboratorio Nacional de Geoquímica y Mineralogía, Sede Regional Sur de Sonora, Instituto Tecnológico de Sonora, Ciudad Obregón, Sonora 85000, México

**Keywords:** Biogeosciences, Infrarred gas analyser, MexFlux, Soil CO_2_ emission, Static chambers, Tropical dry forest

## Abstract

Soil respiration (CO_2_ emission to the atmosphere from soils) is an important component of the global carbon cycle. In highly seasonal ecosystems the magnitudes and the underlying mechanisms that control soil respiration (R_S_) are still poorly understood and measurements are underrepresented in the global flux community. In this dataset, systematic and monthly measurements of R_S_ were conducted with an infrared gas analyzer coupled to a static chamber during 2015, 2016, 2017 and 2019 in a tropical dry forest with a land use history from Northwestern México. These data is useful to assess the intra-annual and seasonal variations of R_S_ at a highly seasonal dry forests and serves as a base line to benchmark soil carbon models in regional and global contexts. The data presented supports the research manuscript: “Soil respiration is influenced by seasonality, forest succession and contrasting biophysical controls in a tropical dry forest in Northwestern Mexico” from Vargas-Terminel et al. [1].

Specifications Table

**Table:** 

Subject	Soil science
Specific subject area	Ecosystem ecology; soil greenhouse gas emissions
Data format	Raw Filtered Analyzed
Type of data	Figure Table
Data collection	Soil respiration data was acquired and analyzed as described in Vargas-Terminel et al. [1]. Briefly, soil respiration was obtained by measuring changes in CO_2_ concentrations with a portable soil flux system attached to a static chamber covering a portion of soil. Each measurement cycle in soil lasted 3 mins and was done monthly during a four-year period in 8 to 12 locations within three sites of tropical dry forest in Northwestern Mexico (a recent abandonment, a mid-secondary forest and an old-growth forest). Also, simultaneous soil temperature and volumetric soil water content measurements were carried with a thermocouple thermometer and a soil moisture sensor, respectively. Finally, soil respiration flux was calculated with commercial soil data processing software.
Data source location	Soil respiration was sampled in tropical dry forest (TDF) from Northwestern México, which depicts a well-defined seasonality due to the influence of the North American Monsoon System in this region [2,3]. The site is located at the Reserva Monte Mojino, a private protected natural area managed by Naturaleza y Cultura Internacional (https://www.natureandculture.org) that relies within the Área de Protección de Flora y Fauna Sierra de Álamos–Río Cuchujaqui in the municipality of Álamos, Sonora [4]. Historically, this site has undergone land-use transformations due to human activities, resulting in a forest succession from land abandonment that transitions from early to secondary and old-growth forests [5].
Data accessibility	Repository name: Zenodo Data identification number: DOI: 10.5281/zenodo.8373128 Direct URL to data: https://zenodo.org/record/8373128
Related research article	M.L. Vargas-Terminel, D. Flores-Rentería, Z.M. Sánchez-Mejía, N.E. Rojas-Robles, M. Sandoval-Aguilar, B. Chávez-Vergara, A. Robles-Morua, J. Garatuza-Payan, E.A. Yépez, Soil Respiration Is Influenced by Seasonality, Forest Succession and Contrasting Biophysical Controls in a Tropical Dry Forest in Northwestern Mexico, Soil Systems. 6 (2022) 75. https://doi.org/10.3390/soilsystems6040075.

## 1 Value of the Data

• Even though, about half of the tropics display a seasonally dry climate [6], there is still an uncertainty about the contribution of soil greenhouse gases from tropical dry forest to ecosystem C balance [7] and the present database contributes to fill this gap.
• The present soil respiration dataset is worthy to reduce the gaps of knowledge in the seasonal, intra-annual and inter-annual variability of tropical dry forest soils under contrasting environmental settings and across forest succession [1].
• This dataset compiles the efforts of monitoring systematically soil respiration in a seasonally dry forest from Northwestern México in order to serve as a baseline information to test, calibrate and validate process-based ecosystem models for water limited regions [8,9].
• Soil respiration from seasonally dry ecosystems in Northwestern México can contribute to the efforts for strengthening soil-atmosphere flux gas research networks such as MexFlux [10] and AmeriFlux [11] and to consolidate the creative commons access to global databases and software such as Continuous SOil Respiration (COSORE) as described in Bond-Lamberty et al. [12] and the Soil Respiration Database (SRDB) reported in Jian et al. [13].
• Assessing the magnitude of soil carbon fluxes in natural protected areas contribute to define the ecosystem potential to serve as a nature climate solution [14].

## 2 Data Description

The dataset contains a four-year measurement period of soil respiration along a forest succession from a seasonally dry forest in the municipality of Álamos, Sonora, México. Climate in the zone is classified as BS1(h’)w [15] with a mean annual temperature of 24°C and a mean annual precipitation of 706 mm [16]. The study area confers a well-defined seasonality that depicts a dry season (November to May) and a wet season, which ∼80% of the total annual precipitation is distributed in this period [17].

**Fig. 1:**
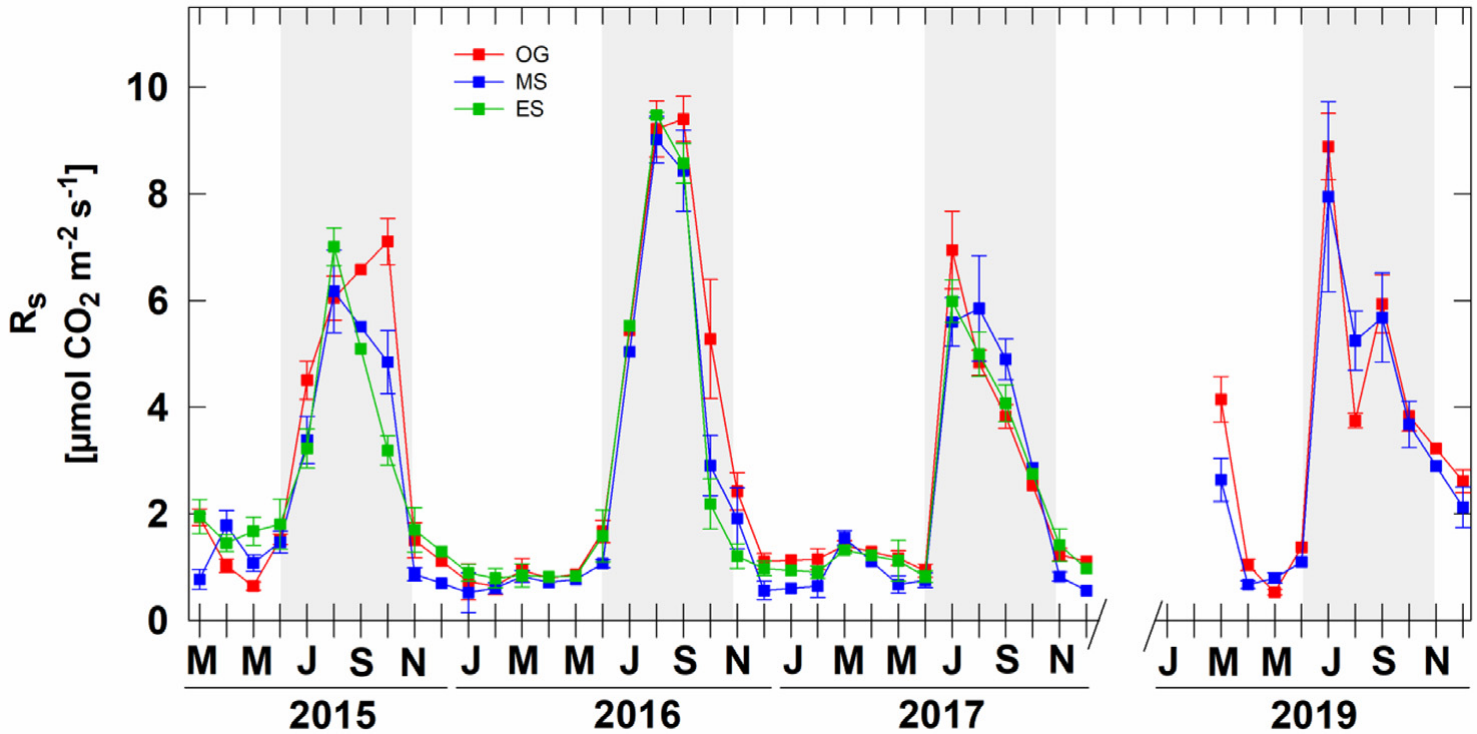
Monthly means of soil respiration (R_S_) in an old-growth (OG), mid-secondary (MS) and early-secondary (ES) tropical dry forest in Northwestern Mexico modified from Vargas-Terminel et al. [1] (see Fig. 1 in the related research article). The data are presented as mean ± standard. Early-secondary (ES) site was not sampled during 2019. Shaded areas show the wet season in the tropical dry forest.

The information located in this repository [18] is described in Table 1, while Fig. 1 shows the monthly mean inter-annual and seasonal variations of soil respiration during 2015, 2016, 2017 and 2019 in an old-growth, mid-secondary and early-secondary tropical dry forests.

**Table 1:** Soil respiration data set description from tropical dry forest in Northwestern Mexico.

Variable	Description	Units
Site	Study site: Álamos, Sonora	Text
Ecosystem	Ecosystem type: tropical dry forest	Text
Forest	Forest succession stage: old-growth, mid-secondary and early-secondary	Text
Year	Year of collection	Number
Month	Month of collection	Number
Season	Season of the year: dry or wet season	Text
Plot	Number of soil collar sampled	Number
R_S_	Soil respiration	µmol CO_2_ m^−2^ s^−1^
TS	Soil temperature	°C
SWC	Volumetric water content	m^3^ m^−3^

## 3 Experimental Design, Materials and Methods

**Fig. 2:**
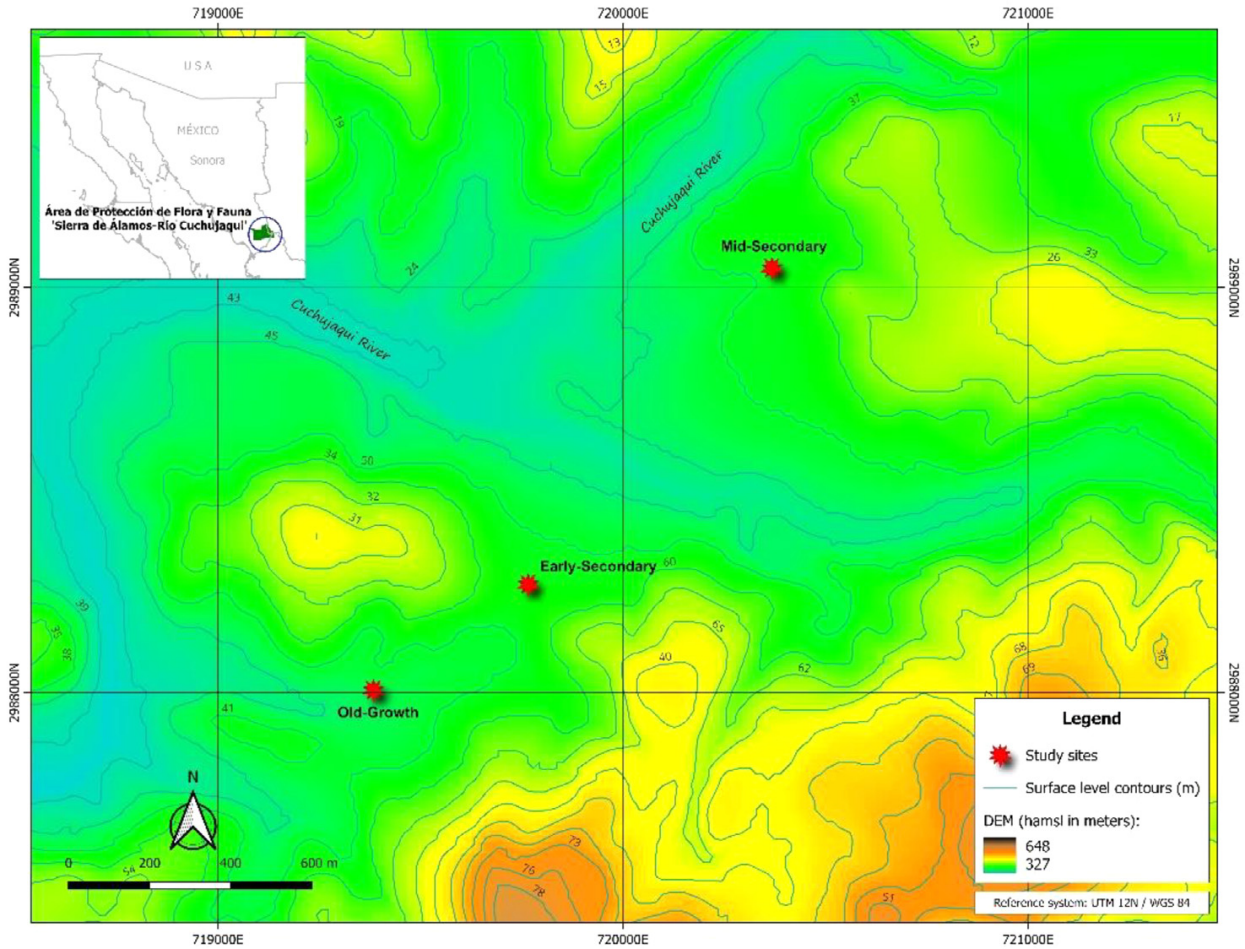
Location of tropical dry forest sampling sites in Northwestern México.

Soil respiration (R_S_) was measured in the northmost limit of the TDF in the Americas. Here, three sites were selected according to the TDF succession pathway in the Pacific Coast of México from Álvarez-Yépiz et al. [5]. The TDF succession sites include an early secondary forest (ES; 26.999202°N, -108.785681°W), which was cleared for intensive livestock and agriculture and abandoned for ∼10 years, followed by a mid-secondary forest (MS, 27.006065°N, -108.779109°W) with a recovery period of ∼40 years since the last land clearing and finally, an old-growth forest (OG, 26.996829°N, -108.789193°W) a well-conserved site that never has been cleared and maintains the representative species composition from TDF. The landscape within the sites shows a mean height above mean sea level (hamsl) of 378 m (Fig. 2).

**Fig. 3:**
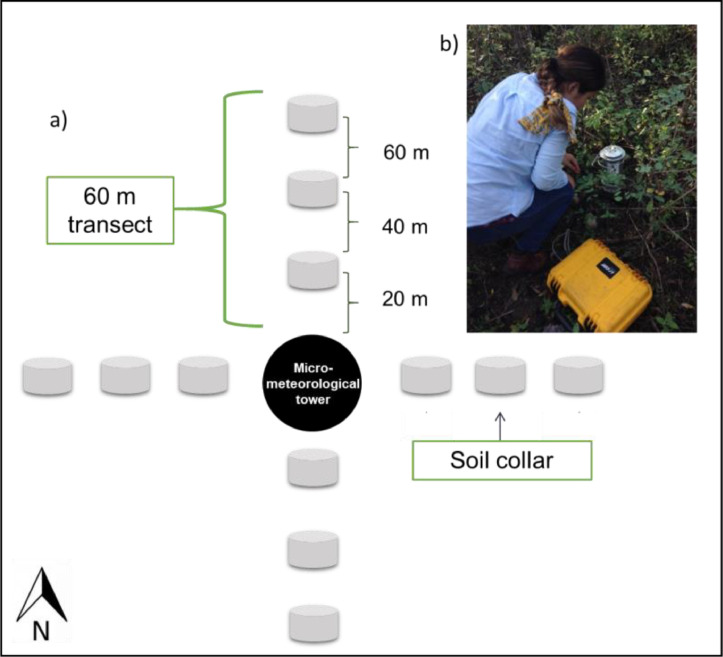
Experimental design for soil respiration (R_S_) sampling collars. Soil collars were installed in permanent 60 m transect distributed along the four cardinal points (a) and R_S_ measurements were conducted monthly during 2015, 2016, 2017 and 2019 with a soil portable infrared gas analyzer (b).

Monthly R_S_ measurements were conducted from March 2015 to December 2017 at OG, MS and ES, while, from March to December 2019 only OG and MS sites were sampled. The experimental design at each site consisted in establishing four permanent transects of 60 m radiating away from a central micrometeorological tower in the direction of each cardinal point, at each transect 10 cm diameter PVC collars were distributed every 20 m (Fig. 3a). Each collar was 8 cm tall and a portion of about 4 cm was buried. R_S_ measurements were conducted using a portable infrared soil gas analyzer (LI-8100, LI-COR Biosciences, Lincoln, Nebraska, USA) connected to a static chamber (model: 8100-102, LI-COR Biosciences, Lincoln, NE, USA) (Fig. 3b). In each soil collar, the CO_2_ concentration was recorded by the analyzer for 180 s and between each measurement a pre-purge and a post-purge were conducted with the aim of removing the trace gas inside the chamber [19]. Also, in an adjacent zone to each soil sampling collar, simultaneous soil temperature and volumetric water content measurements were carried out at 10 cm depth with thermocouple thermometer (Barnant Co., Barrington, IL, USA) and a soil moisture sensor (Theta Probe ML2x, Delta Services, Cambridge, UK). Finally, soil respiration flux rates were calculated using SoilFluxPro^Ⓡ^ (version 4.0.1, LI-COR Biosciences, Lincoln, NE, USA). A detailed information of experimental design and measurements can be found in Vargas-Terminel et al. [1].

## Limitations

In total, 48 soil collars were installed and distributed among four transects; 12 collars within each site. However, in order to avoid drastic changes in environmental conditions during measuring periods, a window of maximum 3 h was scheduled to complete a measurement cycle. Site features (vegetation growth) and access conditions (flooding) during some sampling campaigns precluded the entrance to soil collars in each site. Under these circumstances, a minimum of 8 collars per site were aimed.

## Ethics Statement

This work did not involve human subjects, animals or social media data.

## CRediT authorship contribution statement

**Martha L. Vargas-Terminel:** Conceptualization, Methodology, Formal analysis, Investigation, Data curation, Writing – original draft. **Dulce Flores-Rentería:** Methodology, Formal analysis, Supervision, Writing – original draft. **Zulia M. Sánchez-Mejía:** Methodology, Formal analysis, Supervision, Writing – review & editing. **Nidia E. Rojas-Robles:** Investigation, Writing – review & editing. **Maritza Sandoval-Aguilar:** Investigation, Writing – review & editing. **Bruno Chávez-Vergara:** Methodology, Formal analysis, Supervision, Writing – review & editing. **Agustín Robles-Morua:** Supervision, Writing – review & editing. **Jaime Garatuza-Payan:** Supervision, Writing – review & editing. **Enrico A. Yépez:** Conceptualization, Resources, Supervision, Writing – original draft, Funding acquisition.

## Data Availability

Chronological dataset of soil respiration fluxes from a seasonally dry forest in Northwest México (Original data) (ZENODO)
